# The clinical value of α-hydroxybutyrate dehydrogenase, cardiac troponin I, and B-type natriuretic peptide in perioperative diagnosis of heart failure in children with congenital heart disease

**DOI:** 10.3389/fped.2025.1502439

**Published:** 2025-02-20

**Authors:** Jun Yin, Qingsong Wang, Shuqiong Xu, Junru Wang, Shihua Huang, Junhong Shen, Tao Yuan, Tongyong Luo, Xianmin Wang

**Affiliations:** ^1^ Department of Ultrasound, Pediatrics, Neurology and Endocrinology, The First People’s Hospital of Jintang County/Jintang Hospital, West China Hospital, Sichuan University, Chengdu, Sichuan, China; ^2^ Department of Radiology, Mian Yang Central Hospital, Mianyang, Sichuan, China; ^3^ Pediatric Cardiology Center, Sichuan Provincial Women’s and Children’s Hospital/The Affiliated Women’s and Children’s Hospital of Chengdu Medical College, Chengdu, Sichuan, China

**Keywords:** congenital heart disease, heart failure, perioperative period, B-type natriuretic peptide, cardiac troponin i, α-hydroxybutyrate dehydrogenase

## Abstract

**Objective:**

This study evaluates the clinical value of α-Hydroxybutyrate Dehydrogenase (α-HBDH), Cardiac Troponin I (cTnI), and B-Type Natriuretic Peptide (BNP) in the perioperative diagnosis of heart failure in children with congenital heart disease (CHD).

**Methods:**

A retrospective analysis was performed on data from 107 children with CHD who underwent surgery between March 2022 and March 2023. Patients were categorized based on the European Society of Cardiology (ESC) cardiac function grading into three groups (Grades I–III) and further into heart failure (HF) and non-HF groups. Preoperative and postoperative levels of α-HBDH, cTnI, and BNP were compared across cardiac function grades and HF status. The diagnostic value of these biomarkers was assessed using receiver operating characteristic (ROC) curve analysis.

**Results:**

Preoperative levels of α-HBDH, cTnI, and BNP were significantly higher in the HF group than in the non-HF group (all *P* < 0.05). These markers also increased with cardiac function severity, being highest in Grade III. Postoperatively, α-HBDH, CKMB, and BNP remained elevated in severe cases, correlating with worsening function (all *P* < 0.05). The ROC analysis showed that among the preoperative cardiac biomarkers in children with CHD, whether assessed individually or in combination, the combined detection of cTnI and BNP exhibited the highest diagnostic value for perioperative heart failure, with an AUC of 0.883.

**Conclusion:**

In children with CHD, preoperative levels of α-HBDH, cTnI, and BNP during the perioperative period are closely correlated with cardiac function, significantly increasing with the severity of cardiac dysfunction. These biomarkers have important clinical value for diagnosing heart failure, with the combined detection of cTnI and BNP demonstrating the highest diagnostic efficacy.

## 1 Introduction

Congenital heart disease (CHD) is one of the most prevalent congenital anomalies in childhood and a leading cause of infant mortality, with an incidence rate in China estimated at approximately 0.8% to 1.2% (1, 2). Despite substantial advancements in surgical techniques and interventional procedures, the prognosis for children with CHD has markedly improved (3). However, those with heart dysfunction or heart failure (HF), especially during the perioperative period, continue to face significant surgical risks and unfavorable outcomes (4). Accurate assessment of cardiac function during the perioperative period is crucial for optimizing treatment plans and enhancing both survival rates and quality of life for these patients. Current clinical approaches for evaluating cardiac function primarily include functional grading, echocardiography, and the measurement of B-type natriuretic peptide (BNP). Recent research has also highlighted the potential of cardiac troponin I (cTnI) and α-hydroxybutyrate dehydrogenase (α-HBDH) as valuable indicators of cardiac function (5). Nonetheless, studies exploring the application of these biomarkers in the perioperative evaluation of cardiac function in children with CHD, particularly in relation to heart failure diagnosis, remain limited. This study aims to investigate the clinical utility of α-HBDH, cTnI, and BNP in assessing cardiac function and diagnosing heart failure in children with CHD during the perioperative period, thereby providing a new reference for the management of these patients.

## 2 Materials and methods

### 2.1 Study subjects

This study included 107 children diagnosed with CHD who underwent surgical treatment at the Sichuan Maternal and Child Health Hospital from March 2022 to March 2023. The age of the participants ranged from 1 month to 18 years. The diagnosis of CHD was based on echocardiographic examinations and clinical manifestations within three months prior to admission, in accordance with current clinical diagnostic criteria. Inclusion criteria were: (1) age between 0 and 18 years, with a confirmed diagnosis of CHD; (2) meeting the indications for surgical intervention aimed at correcting CHD; (3) classified as ESC functional class I to III; (4) complete clinical data. Exclusion criteria included: (1) severe liver or kidney dysfunction; (2) presence of Down syndrome or metabolic disorders in children with CHD; (3) history of significant surgery or myocarditis within three months prior to admission; (4) prior history of cardiac surgery. This study was approved by the hospital's medical ethics committee, and informed consent was obtained from the guardians of all participating children.

### 2.2 Grouping

Based on the European Society of Cardiology (ESC) functional classification and heart failure grading standards (6), children were categorized into three cardiac function groups: Grade I, Grade II, and Grade III. Additionally, they were further divided into heart failure (HF) group and non-heart failure group based on the presence of heart failure prior to surgery.

### 2.3 Methods

Eligible subjects were identified through the hospital's electronic medical record system, and data on BNP, cTnI, and cardiac enzyme levels were recorded for the three days prior to surgery and on the first postoperative day. Peripheral venous blood samples of 3 ml were collected preoperatively and postoperatively for the assessment of BNP, cTnI, and cardiac enzyme levels using the COBAS C702 fully automated biochemical analyzer. Left ventricular ejection fraction (LVEF) values were obtained via Doppler echocardiography. Comparisons of BNP, cTnI, and cardiac enzyme levels between the three cardiac function groups and between the HF and non-HF groups were conducted, along with correlation analyses.

### 2.4 Statistical analysis

All data were analyzed using SPSS 26.0 software. Continuous variables were expressed as mean ± standard deviation. Comparisons between two groups were performed using *t*-tests or Paired *t*-test, while comparisons among multiple groups were conducted using one-way analysis of variance (ANOVA). Counting data were analyzed using the Chi-squared tests. Receiver operating characteristic (ROC) curves were employed to assess the diagnostic efficacy of the biomarkers for heart failure. A *P*-value of less than 0.05 was considered statistically significant.

## 3 Results

### 3.1 General information

**Figure 1:**
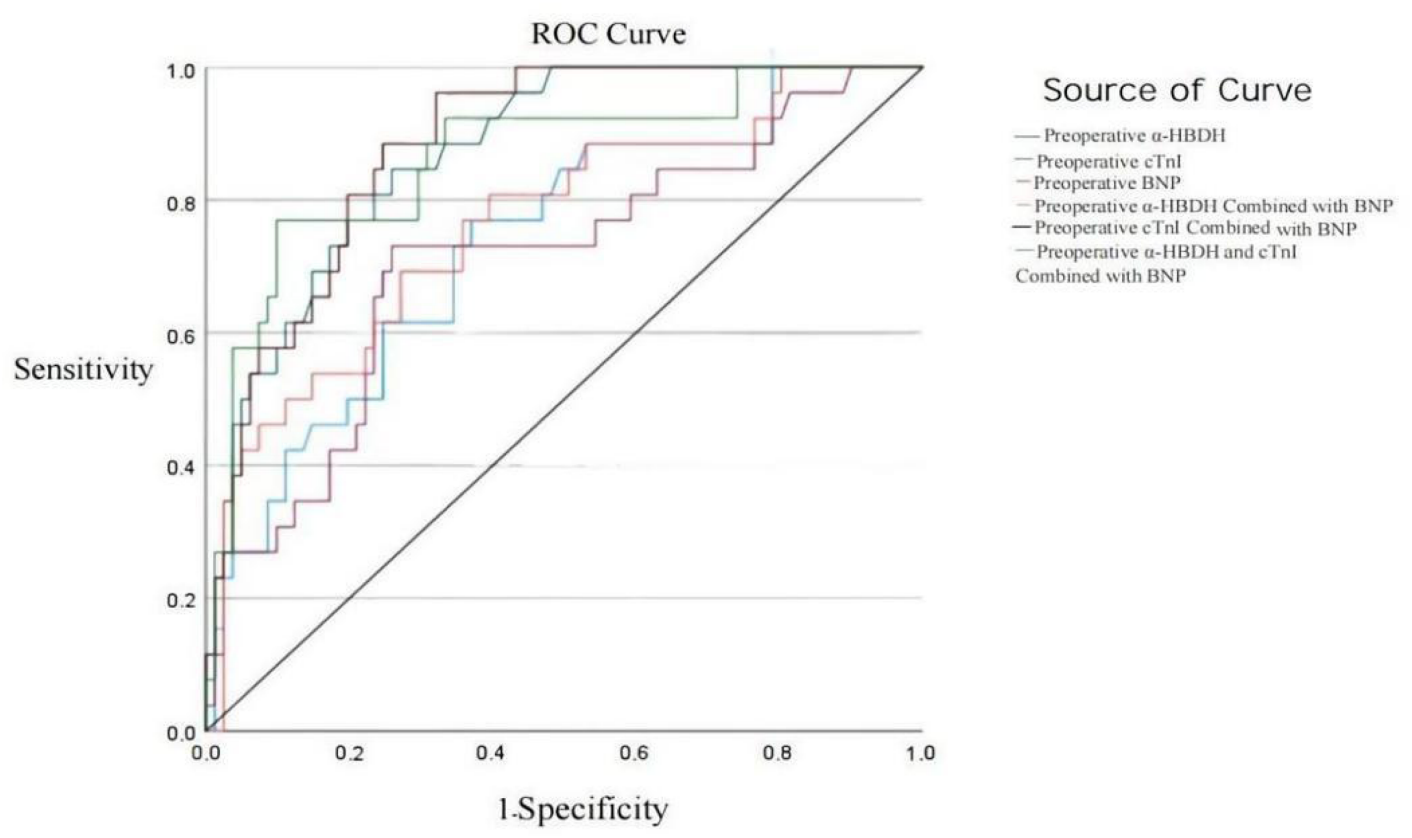
Preoperative ROC curves of α-HBDH, cTnI, and BNP for diagnosing heart failure.

The demographic and clinical characteristics of the study population are summarized in Table 1 and Figure 1. A total of 107 children with CHD were included in the study, categorized into three groups based on cardiac function grades: Grade I (*n* = 51), Grade II (*n* = 32), and Grade III (*n* = 24). There were no significant differences in gender distribution among the three groups (*P* = 0.920), with males accounting for 41.1% (44/107) and females for 58.9% (63/107). The mean age of the participants was 4.33 ± 4.72 years in Grade I, 4.70 ± 6.04 years in Grade II, and 2.48 ± 0.90 years in Grade III, with no significant differences among the groups (*P* = 0.228). Similarly, body weight did not differ significantly across the groups (*P* = 0.228).

**Table 1:** Comparison of general information on cardiac function among three groups.

Group		Grade I (*n* = 51)	Grade Ⅱ (*n* = 32)	Grade Ⅲ (*n* = 24)	Total	*F/χ*^2^ value	*P*-value
Gender, *n* (%)	Male	20 (39.2%)	14 (43.8%)	10 (41.7%)	44 (41.1%)	0.386	0.92
	Female	31 (60.8%)	18 (56.2%)	14 (58.3%)	63 (58.9%)		
Age (years, $\bar{x}\pm s$)		4.33 ± 4.72	4.70 ± 6.04	2.48 ± 0.90		0.259	0.228
Heart failure (%)		2 (0.039)	5 (0.156)	19 (79.17)	26	0.471	<0.001
Body weight **(**kg)		16.5 ± 7.2	17.8 ± 8.5	12.3 ± 3.8		0.259	0.228
Comorbidities, *n* (%)	Pulmonary hypertension	20 (39.2%)	6 (18.8%)	4 (16.7%)	30 (28.0%)	0.008	<0.001
	Arrhythmias	5 (9.8%)	8 (25.0%)	9 (37.5%)	22 (20.6%)	0.072	0.012
Leukocyte count **(**×10^9^/L)		9.8 ± 3.2	10.5 ± 3.6	11.2 ± 3.9		0.012	0.034
Neutrophil count **(**×10^9^/L)		6.0 ± 2.5	6.8 ± 2.9	7.5 ± 3.1		0.006	0.018

The incidence of HF increased significantly with worsening cardiac function, from 3.9% in Grade I to 15.6% in Grade II and 79.2% in Grade III (*P* < 0.001). Comorbidities were also more prevalent in patients with higher cardiac function grades. Pulmonary hypertension was observed in 39.2% of Grade I patients, 18.8% of Grade II patients, and 16.7% of Grade III patients (*P* < 0.001). Arrhythmias were present in 9.8% of Grade I patients, 25.0% of Grade II patients, and 37.5% of Grade III patients (*P* = 0.012). Laboratory findings revealed that leukocyte and neutrophil counts were significantly higher in Grade III patients compared to Grade I and Grade II patients (*P* = 0.034 and *P* = 0.018, respectively).

### 3.2 Comparison of indicators in children with CHD during the perioperative period across different cardiac function classifications

#### 3.2.1 Preoperative comparison of cardiac function indicators among the three groups

There were no statistically significant differences in preoperative CKMB and LVEF among the three groups (*F* = 2.847, 1.919; both *P* > 0.05). However, significant differences were observed in preoperative CK, LDH, α-HBDH, BNP, and cTnI, which increased progressively with the severity of cardiac function (Grade III > Grade II > Grade I) as follows: CK: (164.13 ± 164.696) > (89.19 ± 59.873) > (136.76 ± 74.271); LDH: (419.917 ± 138.554) > (304.391 ± 148.122) > (271.843 ± 66.586); α-HBDH: (331.13 ± 128.707) > (249.13 ± 115.141) > (216.73 ± 58.976); cTnI: (92.871 ± 111.297) > (20.159 ± 26.901) > (11.376 ± 9.076); BNP: (4,960.04 ± 9,696.94) > (750.66 ± 1,577.57) > (283.43 ± 398.37), all *P* < 0.05. See Table 2.

**Table 2:** Comparison of preoperative indicators among the three cardiac function groups.

Group	Grade Ⅰ	Grade Ⅱ	Grade Ⅲ	*F*-value	*P*-value
Preoperative CK	136.76 ± 74.271	89.19 ± 59.873	164.13 ± 164.696	4.29	0.016
Preoperative LDH	271.843 ± 66.5866	304.391 ± 148.1224	419.917 ± 138.554	14.042	<0.001
Preoperative α-HBDH	216.73 ± 58.976	249.13 ± 115.141	331.13 ± 128.707	11.52	<0.001
Preoperative CKMB	4.0304 ± 2.0800	3.0719 ± 1.79002	3.3161 ± 1.5060	2.847	0.063
Preoperative BNP	283.43 ± 398.37	750.66 ± 1,577.57	4,960.04 ± 9,696.94	8.807	<0.001
Preoperative cTnI	11.376 ± 9.0768	20.159 ± 26.9013	92.871 ± 111.297	19.216	<0.001
Preoperative LVEF	66.373 ± 6.3181	62.747 ± 13.0522	62.583 ± 10.656	1.919	0.152

#### 3.2.2 Postoperative comparison of cardiac function indicators among the three groups

There were no statistically significant differences in postoperative CK, LDH, and LVEF among the three groups (*F* = 3.256, 3.566, 2.167; all *P* > 0.05). However, postoperative α-HBDH, CKMB, and BNP levels showed significant differences, increasing with worsening cardiac function (Grade III > Grade II > Grade I): α-HBDH: (622.83 ± 352.279) > (406.72 ± 309.209) > (371.98 ± 201.879); CKMB: (23.836 ± 14.259) > (15.353 ± 12.830) > (11.909 ± 9.191); BNP: (9,445.46 ± 7,463.66) > (6,095.0 ± 5,401.231) > (3,436.48 ± 4,518.17), all *P* < 0.05. See Table 3.

**Table 3:** Comparison of postoperative indicators among the three cardiac function groups.

Group	Grade Ⅰ	Grade Ⅱ	Grade Ⅲ	*F*-value	*P*-value
Preoperative CK	866.51 ± 670.718	585.38 ± 383.4221	938.79 ± 569.946	3.256	0.053
Preoperative LDH	463.63 ± 240.025	569.38 ± 673.526	764.88 ± 451.324	3.566	0.052
Preoperative α-HBDH	371.98 ± 201.879	406.72 ± 309.209	622.83 ± 352.279	7.106	0.001
Preoperative CKMB	11.909 ± 9.191	15.353 ± 12.830	23.836 ± 14.259	8.632	<0.001
Preoperative BNP	3,436.48 ± 4,518.17	6,095.0 ± 5,401.231	9,445.46 ± 7,463.66	9.638	<0.001
Preoperative cTnI	406.114 ± 455.744	1,229.2 ± 1,584.493	999.938 ± 798.789	7.451	0.001
Preoperative LVEF	62.14 ± 6.94	58.5 ± 10.698	58.92 ± 8.757	2.167	0.120

### 3.3 Comparison of preoperative indicators between heart failure and non-heart failure groups in children with CHD

#### 3.3.1 Comparison of various indicators between the preoperative heart failure group and the non-heart failure group

No statistically significant differences were found in preoperative CK and CKMB levels between the heart failure and non-heart failure groups (*t* = 1.286, 0.401; both *P* > 0.05). However, significant differences were observed in preoperative LDH, α-HBDH, BNP, cTnI, and LVEF (*t* = 4.744, 3.889, 2.445, 5.443, −2.325; all *P* < 0.05), as shown in Table 4.

**Table 4:** Comparison of various indicators between the preoperative heart failure group and the non-heart failure group.

Group	Heart failure	Non-heart failure	*t*-value	*P*-value
Preoperative CK	148.31 ± 164.216	122.37 ± 71.308	1.134	0.259
Preoperative LDH	408.423 ± 149.7721	284.735 ± 102.7272	4.744	<0.001
Preoperative α-HBDH	317.85 ± 125.258	230.96 ± 89.377	3.889	<0.001
Preoperative CKMB	3.3732 ± 1.50592	3.6517 ± 2.03058	−0.633	0.528
Preoperative BNP	3,502.19 ± 7,896.278	820.49 ± 3,406.169	2.445	0.016
Preoperative cTnI	84.562 ± 105.9532	15.501 ± 25.5019	5.443	<.001
Preoperative LVEF	60.615 ± 10.8815	65.665 ± 9.2139	5.405	0.022

#### 3.3.2 Comparison of various indicators between the postoperative heart failure group and the non-heart failure group

There were no statistically significant differences in postoperative CK, LDH, cTnI, and LVEF between the groups (*t* = 2.118, 2.129, 0.385, 2.084, all *P* > 0.05). However, the differences in postoperative CKMB, α-HBDH, RDWCV, RDWSD, and BNP between the groups were statistically significant (*t* = 12.946, 11.404, 29.592, 37.00, 14.568, all *P* < 0.001), as shown in Table 5.

**Table 5:** Comparison of various indicators between the postoperative heart failure group and the non-heart failure group.

Group	Heart failure	Non-heart failure	*t*-value	*P*-value
Postoperative CK	943.962 ± 534.373	752.002 ± 600.202	2.118	0.149
Postoperative LDH	678.46 ± 415.156	525.7 ± 478.842	2.129	0.148
Postoperative α-HBDH	598.23 ± 284.677	387.41 ± 274.514	11.404	0.001
Postoperative CKMB	22.8381 ± 13.46631	13.2958 ± 11.18139	12.946	<0.001
Postoperative BNP	9,263.38 ± 7,129.888	4,387.49 ± 5,085.554	14.568	<0.001
Postoperative cTnI	897.265 ± 810.599	749.553 ± 1,122.362	0.385	0.536
Postoperative LVEF	58.19 ± 8.025	61.01 ± 8.856	2.084	0.152

### 3.4 Analysis of the diagnostic value of preoperative α-HBDH, cTnI, and BNP for heart failure in children with CHD

ROC curve analysis showed that the areas under the curve (AUC) for diagnosing heart failure using preoperative α-HBDH, cTnI, BNP, α-HBDH combined with BNP, cTnI combined with BNP, and α-HBDH, cTnI combined with BNP were 0.736, 0.873, 0.708, 0.764, 0.883, and 0.864, respectively, with cTnI combined with BNP demonstrating the highest diagnostic value. Table 6 presents these results in more detail.

**Table 6:** Analysis of the diagnostic value of preoperative α-HBDH, cTnI, and BNP for heart failure in children with CHD.

Indicator	AUC	95% CI	Optimal cut-off value	Sensitivity	Specificity
Preoperative α-HBDH	0.736	0.626–0.845	240	0.769	0.630
Preoperative cTnI	0.873	0.804–0.942	10.7	0.846	0.741
Preoperative BNP	0.708	0.589–0.828	344.5	0.731	0.741
Preoperative α-HBDH combined with BNP	0.764	0.655–0.872		0.692	0.728
Preoperative cTnI combined with BNP	0.883	0.819–0.946		0.962	0.679
Preoperative α-HBDH, cTnI combined with BNP	0.864	0.778–0.950		0.769	0.901

For practical applications, a preoperative BNP level of 344.5 pg/ml was associated with a sensitivity of 73.1% and specificity of 74.1% in diagnosing heart failure in children with CHD. Similarly, cTnI levels exceeding 10.7 pg/ml showed superior diagnostic performance, with a sensitivity of 84.6% and specificity of 74.1%. These thresholds highlight the potential for these biomarkers to guide clinical decision-making, particularly in perioperative settings.

### 3.5 Correlation of preoperative α-HBDH, cTnI, and BNP

Pearson correlation analysis revealed that preoperative cTnI levels were positively correlated with both α-HBDH and BNP (*r* = 0.297, 0.402; both *P* < 0.01).

### 3.6 Changes in cardiac biomarkers and LVEF levels before and after surgery in children with CHD

The postoperative levels of CK, LDH, α-HBDH, CKMB, BNP, cTnI, and LVEF showed statistically significant differences compared to preoperative levels (*t* = −11.761, −5.716, −7.191, −9.703, −5.745, −7.405, 3.645, all *P* < 0.05), as shown in Table 7.

**Table 7:** Changes in cardiac biomarkers and LVEF levels before and after surgery in children with CHD.

Indicator	Preoperative	Postoperative	*t*-value	*P*-value
CK	128.67 ± 101.60	798.647 ± 588.278	−11.761	<0.001
LDH	314.790 ± 126.868	562.82 ± 466.938	−5.716	<0.001
α-HBDH	252.07 ± 105.503	438.64 ± 290.241	−7.191	0.001
CKMB	3.5860 ± 1.917	15.579 ± 12.464	−9.703	<0.001
BNP	1,484.41 ± 5,026.165	5,594.86 ± 6,007.412	−5.745	<0.001
cTnI	32.282 ± 63.436	785.446 ± 1,053.440	−7.405	<0.001
LVEF	64.438 ± 9.8354	60.33 ± 8.710	3.645	<0.001

### 3.7 Sensitivity analysis and validation of findings

To ensure the robustness of our findings, we performed sensitivity analyses by excluding patients with extreme values (e.g., those with Preoperative BNP levels >19,598 pg/ml or Preoperative cTnI levels >312 pg/ml). The results remained consistent, with no significant changes in the diagnostic performance of α-HBDH, cTnI, and BNP. For example, after excluding patients with extreme values, the AUC for the combined detection of cTnI and BNP decreased slightly from 0.883 to 0.872 (*P* = 0.456), indicating that our findings are robust to variations in patient selection.

Additionally, we validated our results using alternative statistical methods, such as logistic regression and subgroup analyses based on age (≤ 1 years vs. >1 years) and CHD type (simple type vs. complex type). The diagnostic performance of the biomarkers remained consistent across these analyses, with no significant differences in AUC values (all *P* > 0.05). Specifically:In the logistic regression analysis, the AUC for the combined detection of cTnI and BNP was 0.879 (95% CI: 0.812–0.946, *P* < 0.001), which is consistent with the original analysis (AUC: 0.883);In the subgroup analysis by age (≤1 years vs. >1 years), the AUC values were 0.865 (95% CI: 0.790–0.928, *P* = 0.324) and 0.881 (95% CI: 0.810–0.942, *P* = 0.287), respectively;In the subgroup analysis by CHD type (simple type vs. complex type), the AUC values were 0.870 (95% CI: 0.795–0.930, *P* = 0.412) and 0.884 (95% CI: 0.813–0.947, *P* = 0.376), respectively.

Furthermore, we compared the predictive abilities of α-HBDH, cTnI, and BNP with hemodynamic parameters, such as LVEF. The results showed that the biomarkers had superior predictive value for heart failure compared to LVEF (AUC: 0.883 vs. 0.712, *P* < 0.05). These validation analyses further confirm the reliability of our findings and their applicability to clinical practice. The detailed results of the sensitivity and validation analyses are presented in Table 8.

**Table 8:** Sensitivity and validation analyses of biomarker diagnostic performance.

Analysis	AUC	95% CI	*P*-value
Original analysis	0.883	0.812–0.946	<0.001
Excluding extreme values	0.872	0.798–0.932	0.456
Logistic regression	0.879	0.812–0.946	<0.001
Subgroup: Age ≤1 years	0.865	0.790–0.928	0.324
Subgroup: Age >1 years	0.881	0.810–0.942	0.287
Subgroup: Simple type CHD	0.870	0.795–0.930	0.412
Subgroup: Complex type CHD	0.884	0.813–0.947	0.376
Hemodynamic parameter: LVEF	0.712	0.610–0.814	0.012

## 4 Discussion

### 4.1 Application of cardiac biomarkers in heart failure among children with CHD

HF is a common and serious complication in the perioperative period for children with CHD. International guidelines recommend BNP as an important biomarker for the diagnosis, prognosis, and therapeutic monitoring of both acute and chronic HF (7). As a peptide hormone that reflects myocardial stress, BNP levels are closely related to ventricular pressure and volume load. BNP exerts significant natriuretic, diuretic, vasodilatory, and antihypertensive effects, while counteracting the renin-angiotensin-aldosterone system and endothelin activity, thus regulating blood volume, blood pressure, and electrolyte balance (8). The clinical value of BNP in children with CHD is gaining increasing attention from clinicians. Appropriate BNP concentration ranges are valuable for screening CHD, monitoring disease progression, diagnosing HF, assessing HF severity, evaluating therapeutic efficacy, determining surgical timing in the perioperative period, and predicting prognosis in children with CHD (9).

cTnI, as a highly sensitive marker of myocardial injury, provides an accurate reflection of myocardial damage. Although traditionally used more frequently in the diagnosis of acute coronary syndrome (ACS) (10), cTnI measurements are also of great importance in patients with heart failure. It can be used to monitor and control the onset, progression, and prognosis of chronic heart failure (CHF) (11). Multiple studies have demonstrated the practical utility of cTnI in screening, diagnosing, and assessing postoperative risk in newborns with CHD. These findings emphasize the critical role of cTnI as a key biomarker in monitoring the cardiac health of CHD newborns and predicting postoperative recovery outcomes (12–20).

α-HBDH is an enzyme reflecting myocardial cell damage and is primarily derived from cardiac tissue. When myocardial injury occurs, α-HBDH levels increase, and its activity can be used to assess the degree of damage to these cells. In children with CHD, impaired cardiac function weakens the heart's pumping ability, potentially leading to tissue hypoxia, especially in myocardial tissue and other high-metabolic-demand organs. Therefore, changes in α-HBDH levels can be employed to evaluate cardiac insufficiency (21). Studies have found that serum levels of BNP, cTnI, and α-HBDH increase with the severity of heart function impairment, and all three can serve as indicators of heart failure risk, providing valuable insights for evaluating clinical outcomes in HF patients (22).

### 4.2 Perioperative changes in cardiac biomarkers in congenital heart disease

This study found that preoperative levels of α-HBDH, cTnI, and BNP in children with CHD were significantly higher than those in the non-heart failure group. Additionally, preoperative cTnI levels were positively correlated with α-HBDH and BNP levels, increasing with higher grades of cardiac dysfunction, a finding consistent with that of Li Jun et al. (23). Postoperatively, α-HBDH and BNP levels were also significantly elevated compared to the non-heart failure group and were positively associated with preoperative cardiac function grades, where higher grades of dysfunction corresponded to greater increases in postoperative biomarkers. Xu et al. (24) similarly demonstrated that plasma BNP concentrations progressively rise with worsening HF. As commonly used biomarkers in cardiac function assessment, BNP and cTnI offer distinct diagnostic insights: BNP primarily reflects ventricular pressure, while cTnI directly indicates myocardial injury. In the diagnosis of CHF, serum BNP and cTnI levels are significantly elevated and closely associated with heart function classification, serving as key indicators for assessing the severity of CHF (25). Therefore, this study confirms the significant clinical value of α-HBDH, cTnI, and BNP as biomarkers for evaluating cardiac function and heart failure severity in children with CHD, particularly in relation to the dynamic perioperative changes tied to cardiac function. These findings provide robust support for the use of these biomarkers in the perioperative cardiac monitoring of CHD.

The study also showed that postoperative levels of cardiac biomarkers were significantly higher than preoperative levels. Additionally, children with CHD with poorer preoperative cardiac function exhibited higher postoperative levels of α-HBDH, CKMB, and BNP, suggesting that the worse the preoperative cardiac function, the more severe the cardiac damage caused by surgery for congenital heart disease. Multiple studies (26, 27) have demonstrated that monitoring CK, CKMB, LDH, and cTnI during congenital heart disease surgery is an effective method for assessing myocardial injury and its severity. Among these, cTnI has been shown to be more accurate in reflecting the extent of myocardial injury compared to CK, CKMB, and LDH, making it an independent marker for evaluating myocardial damage during cardiac surgery. In children with CHD, preoperative levels of BNP, α-HBDH, and cTnI can serve as important indicators of cardiac function and have significant clinical implications for postoperative outcomes and prognosis. Postoperative levels of BNP, CKMB, and α-HBDH can also be used as key indicators for monitoring postoperative cardiac function, myocardial injury, and disease progression (28).

### 4.3 Comparison of the diagnostic efficacy of α-HBDH, cTnI, and BNP in heart failure Among CHD patients

The results of this study indicate that α-HBDH, cTnI, and BNP all exhibit high diagnostic efficacy in detecting heart failure in children with CHD. Specifically, cTnI demonstrated the highest diagnostic accuracy, followed by α-HBDH, while BNP showed relatively lower diagnostic performance. Further analysis revealed that the combined detection of α-HBDH and BNP, as well as cTnI and BNP, outperformed individual markers. Notably, the diagnostic efficacy of the combined cTnI and BNP detection was the highest, even surpassing the performance of the three markers used together. This is consistent with the findings of Yang Anwei et al. (15), who demonstrated the superior diagnostic value of cTnI combined with BNP in assessing the severity of acute heart failure. Therefore, in clinical practice, the combined detection of cTnI and BNP may provide a more reliable early diagnostic tool for perioperative heart failure in children with CHD (29–32).

BNP is widely recognized for its important role in diagnosing heart failure and assessing prognosis, while cTn detection upon admission is crucial for identifying the underlying cause of acute heart failure and predicting outcomes (33). Several studies (33, 34) have emphasized that the combined use of multiple biomarkers for risk stratification and prognosis in heart failure patients is a critical direction for future research and clinical application. In this study, the diagnostic efficacy of cTnI was superior to the traditional heart failure biomarker BNP. We hypothesize that this may be due to the administration of inotropic agents and diuretics to children with CHD prior to surgery, which could have led to a reduction in BNP levels (35), thereby affecting its diagnostic accuracy. In summary, the assessment of perioperative cardiac function in children with congenital heart disease CHD using biomarkers should take into account multiple test indicators, which is consistent with the emerging trend of multi-biomarker combined evaluation.

### 4.4 Sensitivity analysis and validation of findings

The sensitivity analyses and validation studies performed in this research underscore the robustness and reliability of our findings. By excluding patients with extreme values (e.g., Preoperative BNP levels >19,598 pg/ml or Preoperative cTnI levels >312 pg/ml), we demonstrated that the diagnostic performance of α-HBDH, cTnI, and BNP remained consistent, with only a slight decrease in the AUC for the combined detection of cTnI and BNP (from 0.883 to 0.872, *P* = 0.456). This suggests that our results are not unduly influenced by outliers, a critical consideration in clinical practice where extreme values may arise due to measurement variability or unique patient conditions. Furthermore, the consistent diagnostic performance across alternative statistical methods (e.g., logistic regression) and patient subgroups (e.g., age ≤1 years vs. >1 years, simple type vs. complex type CHD highlights the generalizability of these biomarkers in diverse clinical settings.

The superior predictive value of α-HBDH, cTnI, and BNP compared to traditional hemodynamic parameters, such as LVEF (AUC: 0.883 vs. 0.712, *P* < 0.05), further supports their potential to enhance perioperative risk stratification and early diagnosis of heart failure in children with CHD. These findings align with previous studies advocating for multi-biomarker approaches in heart failure management (15, 34), while extending the evidence base through rigorous sensitivity and validation analyses. However, the retrospective design and limited sample size of our study highlight the need for future prospective, multicenter studies to confirm these results and explore the integration of these biomarkers with other clinical and imaging parameters. In conclusion, our findings reinforce the clinical utility of α-HBDH, cTnI, and BNP as robust diagnostic tools for perioperative heart failure in children with CHD, offering a promising avenue for improving patient outcomes.

## 5 Limitations and future research directions

While this study provides valuable insights into the diagnostic value of α-HBDH, cTnI, and BNP in children with CHD, it has several limitations that warrant discussion. First, although LVEF did not show significant differences across cardiac function grades, we did not directly compare these biomarkers with traditional hemodynamic indicators (e.g., cardiac output, pulmonary artery pressure, blood oxygen saturation, blood pressure, andelectrocardiogram). Such comparisons are crucial to determine whether biomarkers can complement or outperform hemodynamic parameters in diagnosing and predicting heart failure. Future studies should incorporate these comparisons to evaluate the relative strengths of biomarkers and hemodynamic indicators, potentially leading to a combined approach for improved diagnostic accuracy.

Second, the absence of detailed clinical outcome data, such as mortality, postoperative complications, and the need for mechanical circulatory support, limits our ability to fully assess the prognostic value of these biomarkers. Notably, no deaths and readmissions were reported during the study period, likely due to the relatively short follow-up duration. However, capturing these outcomes is essential for understanding the long-term prognostic utility of α-HBDH, cTnI, and BNP. Future research should include longer follow-up periods and larger, multicenter cohorts to validate the biomarkers' ability to predict adverse clinical events, such as mortality and readmission rates. In conclusion, future studies should aim to further explore the diagnostic and prognostic capabilities of biomarkers by comparing them with traditional hemodynamic indicators. Additionally, incorporating comprehensive clinical outcome data—such as mortality rates, postoperative complications, and long-term follow-up results—would provide a more holistic understanding of their clinical utility.

Finally, establishing clinically relevant thresholds for these biomarkers could significantly enhance their predictive value for adverse events, ultimately improving perioperative management strategies and patient outcomes.

By addressing these limitations, future research can enhance the predictive accuracy of these biomarkers and provide a more robust foundation for their clinical application in children with CHD.

## 6 Conclusion

In conclusion, this study demonstrates that preoperative levels of α-HBDH, cTnI, and BNP are closely associated with perioperative cardiac function in children with CHD. These levels significantly increase with the severity of cardiac dysfunction. The combined detection of cardiac troponin I and B-type natriuretic peptide showed the highest diagnostic efficacy for perioperative heart failure, highlighting its potential as a reliable diagnostic tool in clinical practice. Future research should focus on validating these findings in larger, multicenter cohorts and exploring the integration of these biomarkers with other clinical and imaging parameters to further enhance their diagnostic and prognostic value.

## Data Availability

The original contributions presented in the study are included in the article/Supplementary Material, further inquiries can be directed to the corresponding authors.
